# Risk factors of meconium-related ileus in very low birth weight infants: patients-control study

**DOI:** 10.1038/s41598-020-60016-3

**Published:** 2020-03-13

**Authors:** Jeik Byun, Ji-Won Han, Joong Kee Youn, Hee-Beom Yang, Seung Han Shin, Ee-Kyung Kim, Hyun-Young Kim, Sung-Eun Jung

**Affiliations:** 1 Department of Pediatric Surgery, Seoul National University College of Medicine, Seoul, South Korea; 2 Department of Surgery, Seoul National University Bundang Hospital, Seongnam, South Korea; 3 Department of Pediatrics, Seoul National University College of Medicine, Seoul, South Korea

**Keywords:** Infant necrotizing enterocolitis, Ileum

## Abstract

Very low birth weight (VLBW) neonates experience various problems, including meconium-related ileus (MRI). This study investigated the risk factors of MRI and surgical MRI in VLBW infants. VLBW neonates admitted to the Neonatal Intensive Care Unit of Seoul National University Children’s Hospital from October 2002 to September 2016 were included in the study. The diagnostic criteria for MRI were a decreased frequency of defecation with intolerable feeding, vomiting, and increased gastric residue (>50%); meconium-filled bowel dilatation in an imaging study; and no evidence of necrotizing enteritis or spontaneous intestinal perforation. Medical MRIs and surgical MRIs were managed through conventional treatment and surgical intervention. Of 1543 neonates, 69 and 1474 were in the patient and control groups, respectively. The risk factors for MRI include low birth weight (BW), cesarean section delivery, fetal distress, maternal diabetes, maternal hypertension, and maternal steroid use. Low BW and fetal distress were independent risk factors for MRI. Compared to the medical MRI group (n = 44), the risk factors for surgical MRI (n = 25) included males, younger gestational age, low BW, and meconium located at the small bowel. Male gender and low BW were independent risk factors for surgical MRI. Low BW and fetal distress were independent risk factors for MRI and male gender and low BW were independent risk factors for surgical MRI. In VLBW neonates, careful attention to the risk factors for MRI could minimize or avoid surgical interventions.

## Introduction

Infants, especially those with very low birth weight (birth weight less than 1.5  kg), may experience various medical problems, including gastrointestinal (GI) problems. Other GI diseases, including necrotizing enterocolitis (NEC), spontaneous intestinal perforation (SIP), focal intestinal perforation (FIP), and meconium-related ileus (MRI), can also occur^1^. Although NEC, SIP, and FIP are mainly related to intestinal perforation, MRI is a disease associated with meconium-induced intestinal obstruction. Since the term MRI was first used by Kubota in 1999 for meconium obstruction without cystic fibrosis (CF), many cases of MRI without CF have been reported^2,3^. The incidence of surgical MRI has increased recently and is similar to that of surgical NEC and FIP^1^. MRI can be managed by medical treatment with a contrast enema. However, several complications, including intestinal perforation, NEC, shock, and occasionally death, have been reported following contrast enema treatment^4,5^.

Hiromi *et al*. described the risk factors of surgical intestinal disorders in VLBW infants and Masaya *et al*. reported them in ELBW infants^1,6^. However, some studies have reported, not only the risk factors of MRI alone but also comparisons between medical and surgical MRI.

The purpose of this study was to investigate the risk factors associated with MRI in VLBW infants and compare their associated factors, especially between the medical and surgical groups.

## Methods

This study was performed on patients with birth weights less than 1.5  kg who were admitted to the Neonatal Intensive Care Unit (NICU) of Seoul National University Children’s Hospital (SNUCH) from October 2002 to September 2016. We retrospectively reviewed the electronic medical records of patients who were diagnosed with MRI, patients who underwent contrast enemas in imaging studies, and patients who underwent surgery and were diagnosed with MRI after surgery. The data included sex, gestational age (GA), birth weight, Apgar scores (a 1  minute and 5  minutes), the method of delivery, fetal distress, twins or triplets, associated cardiac/gastrointestinal conditions, first formula, maternal age, maternal diabetes mellitus (DM) and gestational diabetes mellitus (GDM), maternal hypertension, oligohydramnios, maternal use of intravenous (IV) antibiotics after premature rupture of membrane (PROM), maternal steroid use, age and body weight at the onset of symptoms, and the location of the meconium. The twin or triplet variable was defined as patients born as a twin or triplet. The risk was compared to singlets.

The diagnosis of meconium-related ileus was made on the basis of decreased frequency of defecation with intolerable feeding related to vomiting and increased gastric contents residue (>50%), meconium-filled small bowel or large bowel dilatation observed through ultrasonography or a contrast study with no evidence of NEC, SIP, or FIP.

The treatment policy of SNUCH is that if a patient is suspected of having MRI, ongoing radiograms and abdominal ultrasounds are performed. If there is no evidence of NEC, trophic feeding is maintained. Glycerin enemas are performed four times a day for one or two days. If there is no response to the glycerin enema, a contrast enema is done. The indications for surgical treatment include the lack of response to medical treatment, the necessity to investigate a differential diagnosis of necrotizing enterocolitis, and bowel perforation following a contrast enema.

The definitions of VLBW and ELBW were birth weights of less than 1.5  kg and 1.0  kg, respectively. Patients with underlying conditions of patent ductus arteriosus (PDA), patent foramen ovale (PFO), and atrial septal defect (ASD) were excluded because these are found in most premature neonates.

The patient group was defined as those who were diagnosed with MRI and were managed with either medical or surgical treatment. The control group was defined as the remaining infants, excluding the patient group and NEC, SIP, and FIP patients. Medical MRI was defined as MRI managed with conventional treatment and surgical MRI was defined as MRI managed with surgical intervention, regardless of whether a contrast enema was done. However, when we compared the risk factors between surgical MRIs and medical MRIs, we excluded four cases of surgical MRIs, in which surgical intervention was performed due to bowel perforation after a contrast enema.

Logistic regression analysis was performed to identify the risk factors in the patient and control groups. Each factor was a variable measured before treatment. To examine the distribution of the factors between the patient and control groups, the frequency and percentage are given for categorical variables and mean, standard deviation, median, minimum, and maximum values for continuous variables. All tests were considered statistically significant at *p*-values of* < *0.05. All statistical analyses were performed using SPSS version 21 (IBM Corporation, Sommers, NY, USA). This study was approved by the Institutional Review Board (IRB) of Seoul National University Hospital (IRB number 1707-043-868). All experiments on humans and/or the use of human tissue samples were performed in accordance with relevant guidelines and regulations with approval of the institutional and/or licensing committee. Informed consent to participate in the study was obtained from a parent and/or legal guardian.

## Results

**Figure 1:**
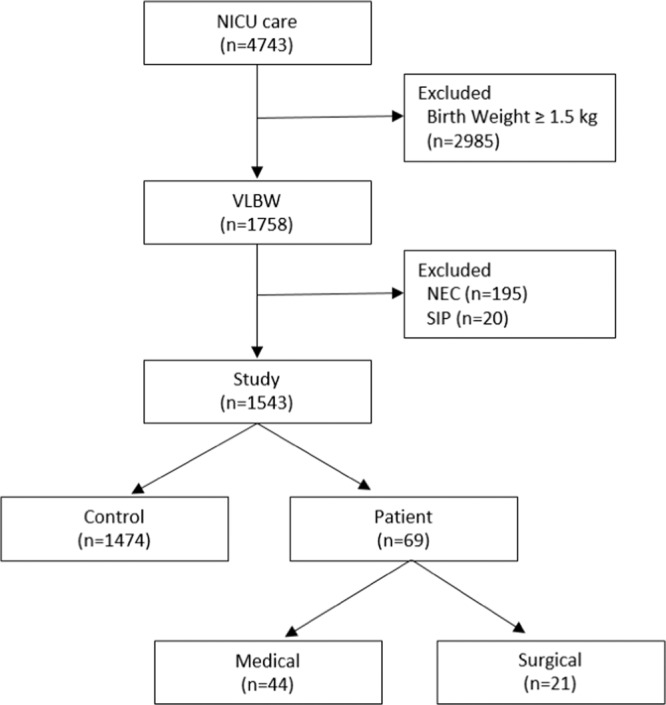
Study diagram.

Among 4743 patients who were admitted to the NICU, excluding NEC (n = 195), SIP (n = 20), and patients with birth weights ≥ 1.5  kg (n = 2985), a total of 1543 neonates were included in the study (Fig. 1). There were 1474 patients in the control group and 69 (4.47%) in the patient group. Males represented 50.71% of the groups and the mean GA and mean birth weights were 28.6 ± 8.0 weeks and 1012 ± 310  g, respectively.

We identified the factors associated with MRI compared to the control group using univariate and multivariate analyses (Table 1). In the univariate analysis, low birth weight, ELBW, cesarean delivery, fetal distress, associated GI conditions, maternal DM, maternal HTN, and maternal steroid use were factors associated with MRI. Multivariate analysis showed that low birth weight (OR = 0.998 (95%CI: 0.997–0.999), *p < *0.001), fetal distress (OR = 3.517 (95%CI: 1.913–6.466), *p < *0.001), maternal DM (OR = 4.026 (95%CI: 1.644–9.860), *p = *0.002), and maternal steroid usage (OR = 4.618 (95%CI: 2.117–10.075), *p < *0.001) were significant factors associated with MRI.

**Table 1:** Factors associated with MRI (Univariate/Multivariate analysis).

Variables	Total (N = 1543) (%)	Univariate analysis			Multivariate analysis	
		Control (N = 1474)	Patients (N = 69)	*P*-value	OR (95%CI)	*P*-value
Sex				0.221		
Male	783 (50.71)	743 (50.41)	40 (57.97)			
Female	760 (49.22)	731 (49.59)	29 (42.03)			
GA (week, mean ± SD)	28.6 ± 8.0	28.58 ± 3.21	28.19 ± 2.48	0.214		
<28	842	800 (54.46)	42 (60.87)	0.609		
≥28	696	669 (45.54)	27 (39.13)			
Birth weight	1012 ± 309	1019 ± 310	858 ± 266	< 0.001	0.998 (0.997–0.999)	< 0.001
SGA				0.365		
No	804 (52.1)	765 (63.7)	39 (58.21)			
Yes	464 (45.23)	436 (36.3)	28 (41.79)			
ELBW	746 (48.7)	695 (47.15)	51 (73.91)			
VLBW	787 (51.3)	769 (52.53)	18 (26.09)	< 0.0001		
Apgar score (mean ± SD)						
1 minute	4.06 ± 2.22	4.09 ± 2.22	3.59 ± 2.07	0.073		
5 minutes	6.13 ± 2.04	6.15 ± 2.04	5.83 ± 2.01	0.201		
Delivery						
Vaginal	518 (34.06)	507 (34.92)	11 (15.94)	0.002	1	0.376
Cesarean section	1003 (65.94)	945 (65.18)	58 (84.06)		1.412 (0.658–3.032)	
Fetal distress						
No	324 (56.3)	315 (59.2)	9 (20.9)	< 0.001	1	< 0.0001
Yes	251 (43.7)	217 (40.8)	34 (79.1)		3.517 (1.913–6.466)	
Twin or triplet	733 (51.84)	704 (52.34)	29 (42.03)	0.097		
Underlying cardiac condition						
No	1153 (84.5)	1092 (84.19)	61 (89.71)	0.226		
Yes	212 (15.5)	205 (15.81)	7 (10.29)			
Underlying GI condition						
No	1290 (77.9)	925 (70.66)	68 (100)	< 0.0001		0.993
Yes	365 (22.1)	365 (29.34)	0 (0)			
Formula				0.567		
BM	490 (45.8)	455 (37.51)	35 (61.40)			
PM	422 (39.4)	422 (34.79)	0 (0)			
Mixed	158 (14.8)	336 (27.70)	22 (38.60)			
Maternal age (year, mean ± SD)	32.86 ± 4.02	32.89 ± 3.92	32.78 ± 3.91	0.822		
Maternal DM						
No	1198 (98.0)	1138 (94.68)	60 (88.24)	< 0.001	1	0.002
Yes	25 (2.0)	18 (1.5)	7 (10.29)		4.026 (1.644–9.860)	
Maternal HTN						
No	1016 (79.3)	971 (79.98)	45 (66.18)	0.007	1	0.745
Yes	266 (20.7)	243 (20.02)	23 (33.82)		1.110 (0.592–2.079)	
Maternal use of steroid						
No	432 (35.9)	424 (37.26)	8 (11.94)	0.001	1	< 0.001
Yes	773 (64.1)	714 (62.74)	59 (88.06)		4.618 (2.117–10.075)	
Maternal use of IV anti after PROM						
No	143 (26.5)	120 (25.26)	23 (35.94)	0.075		
Yes	396 (73.5)	355 (74.74)	41 (64.06)			
Oligohydramnios	249 (21.16)	232 (20.9)	17 (25.37)	0.340		

ELBW, extremely low birth weight; VLBW, very low birth weight; C/sec, cesarean section; BPD, bronchopulmonary dysplasia; GI, gastrointestinal; ROP, retinopathy of prematurity; SD, standard deviation; DM, diabetes mellitus; HTN, hypertension.

In the MRI group, 44 patients were treated conventionally and 25 patients underwent surgery. In the analysis of the risk factors, the medical group (n = 44) was compared to the surgical group (n = 21). In the univariate analysis, male gender, younger GA, ELBW, low birth weight, and location of the meconium at the small bowel on ultrasonography were significantly associated with surgical MRI. Male gender and low birth weight were associated with surgical MRIs in the multivariate analysis (Table 2).

**Table 2:** Factors associated with medical MRI vs. surgical MRI (Univariate/Multivariate analysis).

Variables	Univariate analysis			Multivariate analysis	
	Medical (N = 44) (%)	Surgical (N = 21) (%)	*P*-value	OR (95%CI)	*P*-value
Sex					
Male	22 (50)	17 (81)	0.017	7.077 (1.301–38.505)	0.024
Female	22 (50)	4 (19)		1	
GA (week, mean ± SD)	28.89 ± 2.18	26.52 ± 2.60	< 0.001		0.057
< 28	16 (36.4)	15 (71.4)	0.008		
≥ 28	28 (63.6)	6 (28.6)			
ELBW	28 (63.64)	20 (95.2)	0.007		
VLBW	16 (36.36)	1 (4.8)			
Apgar score					
1 minutes	3.86 ± 2.04	3.12 ± 2.07	0.153		
5 minutes	6.16 ± 1.84	5.24 ± 2.20	0.073		
Birth weight	918 ± 276	713 ± 209	0.002	0.995 (0.990–0.999)	0.027
Delivery					
Vaginal	6 (13.64)	4 (19)	0.575		
Cesarean section	38 (86.36)	17 (81)			
SGA	15 (34.1)	9 (42.6)	0.493		
Fetal distress	24 (57.14)	7 (33.3)	0.124		
Twin or triplet	17 (38.64)	10 (47.6)	0.492		
Underlying cardiac condition					
No	42 (95.45)	19 (79.17)	0.052		
Yes	2 (4.55)	5 (20.83)			
Underlying GI condition					
No	44 (100)	24 (100)	N/A		
Yes	0 (0)	0 (0)			
Formula					
BM	25 (65.79)	8 (38.1)	0.315		
Mixed	13 (34.21)	7 (33.3)			
Maternal age (year, mean ± SD)	32.52 ± 3.91	31.29 ± 8.22	0.412		
Maternal DM	7 (15.9)	1 (4.8)	0.194		
Maternal HTN	17 (39.53)	7 (33.3)	0.101		
Maternal use of steroid	36 (85.71)	19 (90.5)	0.596		
Maternal IV anti	23 (56.1)	19 (90.5)	0.149		
Oligohydramnios	10 (23.81)	7 (33.3)	0.569		
Age at onset of symptom (day, mean ± SD)	4.13 ± 3.18	7.38 ± 9.287	0.122		
Body weight at onset of symptom	878.38 ± 266.48	754.67 ± 271.24	0.146		
Location of meconium on USG					
Small bowel	21 (55.26)	15 (71.4)	0.041	1.991 (0.358–1.068)	0.432
Large bowel	17 (44.74)	3 (14.3)		1	

GA, gestational age; SD, standard deviation; ELBW, extremely low birth weight; VLBW, very low birth weight; RDS, respiratory distress syndrome; BPD, bronchopulmonary dysplasia; ROP, retinopathy of prematurity; USG, ultrasonography.

In the control group, 153 patients died within discharge. Cardiovascular events were the most common cause of death (n = 59), followed by respiratory problems (n = 51), and infections (n = 30). Other causes included perinatal distress, renal failure, fetal hydrops, cerebral infarction, hypovolemic shock, and brain hemorrhage. There were no deaths in the MRI group.

## Discussion

In the past, when a meconium plug was located in the colon and symptoms were relieved after the meconium was excreted following conventional treatment, it was called meconium plug syndrome (MPS). When meconium was sticky and located in the small intestines with poor response to conventional treatment, consequently requiring surgical intervention, it was generally called meconium disease (MD)^6^. However, in 1999, a report by Kubota *et al*. proposed that the term MRI should encompass both MPS and MD^2^. They explained that MRI results from the immature or ineffective peristalsis of the fetal intestine combined with excessive water absorption, producing meconium plugs or sticky meconium in the small bowel and right colon^6^.

A few studies have suggested a risk for MRI in low birth weight infants. In a study by Emil, maternal hypertension, maternal MgSO_4_ therapy, cesarean delivery, and maternal DM were associated with the pathogenesis of MD in ELBW neonates^2,7^. Yamoto *et al*. reported that intrauterine growth retardation was a significant risk factor for surgical MRI in ELBW infants^8^. Okuyama *et al*. reported that twin pregnancies, PROM, and the use of maternal steroids were significant risk factors for surgical MRIs in VLBW infants^1^. In this study, low birth weight, fetal distress, maternal DM, and the maternal use of steroids were independent risk factors for MRI.

MRI perforation is associated with immature or uncoordinated bowel movement, which results in increased intraluminal pressure and perforation^6^. Greenholz *et al*. explained that prematurity itself is sometimes accompanied by bowel perfusion impairment, resulting in bowel dysmotility^9^. The microscopic changes of mucoviscidosis could result from the failure of the distal progression of hepaticopancreatic secretions combined with postnatal bacterial overgrowth, which could provide histological support for stasis as the underlying pathology^9^. As motility returns, the obstructive symptoms appear^9^.

A few studies have explained the association between fetal distress and MRI. There is a report on NEC using umbilical artery perfusion, although it is not about MRI. Anita *et al*. reported that fetal Doppler imaging of the umbilical artery and vein could predict NEC in preterm neonates. As the fetus adapts to the hemodynamic stress of placental insufficiency, blood flow to the splanchnic beds could become insufficient. Therefore, they explored the mesenteric arterial blood flow and umbilical arterial blood flow as predictors of NEC. In their study, although placental disease predisposed to severely growth-restricted NEC neonates, it was not the predominant risk factor for NEC^10^.

Several studies have suggested that maternal DM is a risk factor for MRI^2,7^. However, few studies have explained the exact reason for the finding. However, 40–50% of patients with maternal diabetes have been reported to have neonatal small left colon syndrome^11–13^. Among the babies of diabetic mothers, narrowing of the left colon observed in the barium enemas of neonates with no specific GI symptoms has been seen in some cases and some patients underwent surgery due to bowel perforation. However, the precise mechanism of why neonatal small left colon syndrome occurs in women with diabetes mellitus is unknown.

Few studies have specifically addressed the impact of maternal steroids on MRI patients. In a study by Yamoto, that reported the risk factors for surgical intestinal disorders in ELBW infants, steroids were not associated with MRI but were a risk factor for FIP^8^. In contrast to the results of this study, the use of maternal steroids was reported as a protective factor for MRI (OR = 0.38) in a study by Okuyama. Although there is no explanation for the exact mechanism, it is thought that maternal steroid use increases the GI motility of VLBW patients and has a protective effect^1^. In a study by Sudha that analyzed the factors associated with stool passage by neonates, the newborns born to mothers with betamethasone showed a significantly early first stool passage time. This is also explained by the fact that betamethasone increased GI motility and enzyme induction^14^.

Preterm male infants are known to have significant disadvantages in terms of morbidity and mortality including RDS, BPD, IVH, and GI problems, compared to female infants of similar GA. In a study by Ito *et al*. on the sex differences in mortality and morbidity of preterm infants, male infants, especially those born at gestation age 23–25 weeks had worse gastrointestinal outcomes than females. However, the mechanism of the sex-specific pattern is not yet fully understood^15^.

This study had limitations. First, this was a retrospective study in which the researcher relied on medical records. For more accurate results, a prospective study of MRI is needed in the future. Second, some of the newborns were born in outside hospitals, which resulted in a loss of data. Third, the exact reason for the association between risk factors and MRI could not be identified in this study. Based on this study, further studies to clarify the exact cause of MRI are needed. Fourth, there was a limitation in the selection of control group patients. The control group was also patients admitted to the NICU, so they might have had other underlying diseases, even if they were not NEC or MRI. However, in studies of VLBW neonates, it was difficult to get a control group that is not in the NICU.

In conclusion, the independent risk factors for MRI included lower birth weight, fetal distress, maternal DM, and the maternal use of IV steroids. The independent risk factors for surgical MRI were male gender and lower birth weight. In patients with those risk factors, the possibility of MRI should be considered and surgical and early interpretation and intervention are important. In VLBW neonates, careful attention to the risk factors for MRI could minimize or avoid surgical interventions.
